# Effects of *Acanthus ebracteatus* (Sea Holly) Aqueous Extract as a Functional Feed Additive on Growth Performance, Immune Responses, and Hepatopancreatic Histology in Pacific White Shrimp (*Litopenaeus vannamei*)

**DOI:** 10.3390/ani16121842

**Published:** 2026-06-15

**Authors:** Wassana Prisingkorn, Pattama Wiriyapattanasub, Prasoborn Rinthong, Phadet Hongmanee, Sutee Wongmaneeprateep, Jariyavadee Suriyaphan, Apichet Pholoeng, Worapat Horjarlearn, Kanjana Thumanu, Kanokwan Kamkajon, Eakapol Wangkahart

**Affiliations:** 1 Aquatic Animal Disease and Molecular Biology Laboratory, Department of Fisheries, Faculty of Agriculture, Khon Kaen University, Khon Kaen 40002, Thailand; wasspr@kku.ac.th (W.P.); pattawi@kku.ac.th (P.W.); suthwo@kku.ac.th (S.W.); 2 Pharmaceutical Chemistry and Natural Products Research Unit, Faculty of Pharmacy, Mahasarakham University, Maha Sarakham 44150, Thailand; prasoborn.r@msu.ac.th; 3 Technician and Marketing Support Department, Thaiunion Feedmill PCL, Samut Sakhon 74000, Thailand; boyd8258@yahoo.com; 4 Department of Aquatic Science, Faculty of Science, Burapha University, Chonburi 20131, Thailand; jariyavadee@buu.ac.th; 5 Laboratory of Fish Immunology and Nutrigenomics, Applied Animal and Aquatic Sciences Research Unit, Division of Fisheries, Faculty of Technology, Mahasarakham University, Khamriang Sub-District, Kantarawichai, Maha Sarakham 44150, Thailand; apichet2606254086@gmail.com; 6 Department of Fisheries, Inland Fisheries Research and Development Division, Khonkaen Inland Fisheries Research and Development Center, Khon Kaen 40250, Thailand; ohm_room69@hotmail.com; 7 Synchrotron Light Research Institute (Public Organization), Nakhon Ratchasima 30000, Thailand; kanjanat@slri.or.th; 8 School of Animal Technology and Innovation, Institute of Agricultural Technology, Suranaree University of Technology, Nakhon Ratchasima 30000, Thailand; numfon.001@gmail.com

**Keywords:** antioxidant activity, bioactive feed additive, hepatopancreas histology, immunomodulation, phytogenic compounds, shrimp aquaculture

## Abstract

Intensive shrimp aquaculture has led to increased disease outbreaks and a greater reliance on antibiotics, driving the need for sustainable alternatives. This study examined the phytochemical profile, antioxidant activity, and effects of *Acanthus ebracteatus* (AC) aqueous extract as a natural feed additive on growth performance, and health in Pacific white shrimp (*Litopenaeus vannamei*). Phytochemical analysis of AC aqueous extracts revealed high concentrations of phenolics (162.1 ± 11.78 mg GAE/g), flavonoids (75.8 ± 2.32 mg RE/g), tannins (38.4 ± 0.78 mg TAE/g), and verbascoside (0.48 ± 0.01% *w*/*w*). Strong antioxidant capacity was confirmed with DPPH IC_50_ of 42.6 ± 11.78 µg/mL, ABTS IC_50_ of 2.93 ± 0.02 mg/mL, and FRAP value of 1.41 ± 0.08 mmol FeSO_4_/g extract. A feeding trial was conducted over eight weeks using 300 shrimp randomly assigned to four treatments: control diet and diets supplemented with 1%, 2%, and 3% AC aqueous extract. AC 2% supplementation showed optimal results for growth performance, including improved final body weight, weight gain, and feed conversion ratio. AC 3% treatment maximally enhanced immune responses, significantly elevating catalase, lysozyme, total antioxidant capacity, and hemocyanin, and glutathione peroxidase activity showed a numerical increase in AC-supplemented groups, particularly at the 3% inclusion level, although the differences were not statistically significant. AC 2% supplementation also improved hepatopancreatic histology through oxidative stress modulation and enhanced lipid metabolism. This study provides a comprehensive evaluation of AC aqueous extract by integrating phytochemical characterization, antioxidant profiling, and in vivo assessment of growth, immune response, and hepatopancreatic morphology in Pacific white shrimp.

## Simple Summary

Shrimp farming is an important global industry, but it faces serious challenges from disease outbreaks and the overuse of antibiotics. This study explored the use of a natural plant, *Acanthus ebracteatus* (sea holly), as a feed additive to improve shrimp health in a more sustainable way. We found that this plant extract contains beneficial compounds with strong antioxidant properties. When added to shrimp feed, it improved growth performance, immune responses, and the health of the hepatopancreas (a key organ for digestion and metabolism). In particular, a 2% inclusion level promoted the best growth and tissue health, while a 3% level enhanced immune activity. These findings suggest that sea holly extract could be a promising natural alternative to antibiotics, helping to support healthier and more sustainable shrimp aquaculture.

## 1. Introduction

Aquaculture has experienced continuous rapid global growth, with Pacific white shrimp (*L. vannamei*) emerging as the most extensively cultivated animal species in terms of individual numbers. Approximately 6.3 million tons, equivalent to an estimated 300 to 620 billion individuals, are farmed annually, underscoring its dominant role in global aquaculture. Furthermore, global production has demonstrated a rapid and consistent upward trend, increasing by an average of 300,000 tons per year over the past two decades [1]. Thailand is internationally recognized as one of the foremost producers in the aquaculture sector, with a particular emphasis on the cultivation of Pacific white shrimp. This species has become the cornerstone of Thailand’s shrimp industry due to its adaptability to varying salinity levels, rapid growth rates, and favorable organoleptic properties, making it highly suitable for intensive farming systems. In response to the persistent demands of the aquaculture industry, substantial efforts have been dedicated to the development of genetically improved Pacific white shrimp strains exhibiting enhanced growth performance, improved disease resistance, and a reduced dependence on antimicrobial treatments [2,3]. The indiscriminate use of antibiotics in aquaculture operations continues to contribute to the emergence and proliferation of drug-resistant pathogens [4,5]. A major constraint to shrimp aquaculture in recent years has been the emergence of Early Mortality Syndrome (EMS), caused by *Vibrio parahaemolyticus*, which has severely affected production levels following its first documented occurrence in 2012 [6]. This disease, along with increasing environmental variability, has led to fluctuations in post-larval quality and a decline in optimal farming conditions, contributing to reduced production volumes. While Thailand’s annual shrimp production reached approximately 270,000 tons in 2024 [7], this figure reflects ongoing struggles with disease management and climate-related challenges. Nevertheless, Thailand has maintained its status as a major Pacific white shrimp exporter through strategic implementation of genetic improvement programs, stringent health management strategies, and adoption of environmentally responsible farming methods. Recent breakthroughs in specialized feed formulations have shown considerable promise in reducing disease severity and enhancing shrimp development rates. Functional feed additives are substances incorporated into aquafeeds in small quantities to serve various functions, including enhancing nutritional value, stimulating the immune system, and promoting growth, along with maintaining water quality [8]. Up to date, herbal extracts, derived from plants, have emerged as promising feed additives in the aquafeed industry and are increasingly utilized as prophylactic alternatives to conventional strategies for enhancing production efficiency and supporting the long-term sustainability of shrimp cultures [9,10,11].

Pacific white shrimp (*L. vannamei*) is one of the most economically significant crustacean species and ranks among the most widely cultured shrimp globally [12]. Its high economic value, rapid growth rate, exceptional survival rates under high-density conditions, and notable disease tolerance make Pacific white shrimp a preferred species for intensive grow-out production systems [13]. Recent innovations in shrimp nutrition have highlighted the benefits of incorporating natural substances such as microalgae, amino acids, vitamins, and herbal extracts into feed formulations [14]. These natural additions have demonstrated the ability to boost growth rates, strengthen immune defenses, and improving resistance against *Vibrio* pathogens. Additionally, plant-based compounds and other natural products are increasingly recognized as effective tools for enhancing survival during the early stages of shrimp culture [15]. Owing to their widespread availability, cost-effectiveness, and practical applicability, these natural products have demonstrated effectiveness in enhancing farm productivity while aligning with the sustainability goals of Thailand’s aquaculture sector, as well as ensuring consumer safety and global accessibility. Marine coastal ecosystems harbor numerous plant species with potential applications in aquaculture nutrition and health management, presenting opportunities for the development of natural bioactive compounds [16]. In recent years, increasing attention has been directed toward the investigation of bioactive compounds derived from marine medicinal plants, with the objective of developing dietary supplements and alternative pharmaceutical agents of aquatic animal.

*Acanthus ebracteatus* (AC), commonly known as sea holly, is a coastal medicinal plant widely distributed in Trat Province, Thailand. Previous chemical investigations on this plant revealed the presence of bioactive components including phenolic compounds, aliphatic alcohol, aliphatic glycosides, phenolic glycosides, terpenes, megastigmane glycosides, flavonoids, and lignan glycosides [17,18,19,20]. Each class of these secondary metabolites contributes distinct biological activities relevant to shrimp health: phenolic compounds and flavonoids serve as primary antioxidants that scavenge reactive oxygen species and modulate immune signaling pathways; phenolic glycosides such as verbascoside exhibit potent anti-inflammatory and immunostimulatory effects; terpenes confer antimicrobial activity against bacterial and fungal pathogens; and lignan glycosides and megastigmane glycosides contribute to overall immunomodulatory and cytoprotective effects. These secondary metabolites have collectively exhibited significant pharmacological activities, particularly anti-inflammatory properties [21,22], antioxidant and broad-spectrum antimicrobial efficacy against bacterial and viral pathogens [23]. The substantial presence of polyphenolic components in the AC extracts might be directly related to their antibacterial properties [24]. However, the inclusion of plant extracts in the diets of shrimp has been shown to confer various health benefits, including stress mitigation, growth enhancement, appetite stimulation, immunomodulation, regulation of the gut microbiota, and antimicrobial effects, while also enhancing the amino acid and fatty acid profiles of shrimp muscle and serving as an additional nutritional source in aquafeed [25,26,27]. In the present study, stems and leaves of AC were selected over roots on the basis of their documented richness in phenolics, flavonoids, and verbascoside [20,24], their greater ecological sustainability for harvesting from mangrove habitats, and their practicality for large-scale aquafeed production, given that aerial parts can be collected repeatedly without uprooting or destroying the plant.

Despite the documented pharmacological properties of AC, information regarding the effects of its stem and leaf aqueous extract as a dietary supplement in Pacific white shrimp remains limited. We hypothesized that dietary supplementation with AC aqueous extract would enhance growth performance, antioxidant status, immune-related responses, and hepatopancreatic health in Pacific white shrimp. Therefore, the objectives of this study were to (1) characterize the phytochemical composition and antioxidant properties of the aqueous extract, (2) evaluate its effects on growth performance and feed utilization, (3) assess antioxidant and immune-related parameters, and (4) examine hepatopancreatic morphology and biochemical alterations using histological and SR-FTIR analyses. To our knowledge, this is the first study evaluating AC extract as a dietary supplement in Pacific white shrimp combining phytochemical characterization with in vivo growth, immune, and histological assessments.

## 2. Materials and Methods

### 2.1. Ethics Statement

All healthy Pacific white shrimp were handled, and all experimental procedures were conducted in accordance with the guidelines for the care and use of animals for scientific purposes established by the National Research Council of Thailand (NRCT) and the Institute for the Development of Animals for Scientific Purposes (IAD), Bangkok, Thailand (U1-08206-2562). Animal experiments were conducted under the approval of Khon Kaen University (IACUC-KKU-54/67; Reference No. 660201.2.11/481 (61)).

### 2.2. AC Extract Preparation

The extraction and phytochemical analysis of AC were carried out using two analytical approaches, encompassing both plant extraction and extract preparation procedures. In brief, for the plant extraction process, samples of AC were collected from natural mangrove areas in Trat and Rayong Provinces, Thailand. Healthy white flowered specimens were randomly harvested from the mangrove forest at Paknam Phang Rad (12°41′35″ N, 101°46′56″ E), located within the Rayong Botanical Garden, Klaeng District, Rayong Province, Thailand. Scientific identification was confirmed using the Flora of Thailand, and voucher specimens (Herbarium No. BUU01-07) were deposited at the Department of Marine Science, Faculty of Science, Burapha University. The stems and leaves were cleaned, cut, and dried at 60 °C for 72 h. The dried materials were then ground and sieved (mesh No. 14) before being stored in a closed container until extraction. For aqueous extraction, 100 g of dried powder was mixed with 1 L of deionized water (solid-to-solvent ratio 1:10, *w*/*v*). The mixture was heated at approximately 95–100 °C and maintained under continuous decoction for 30 min. Deionized water was used without pH adjustment. After extraction, the mixture was cooled to room temperature and filtered through Whatman No. 1 filter paper using a Büchner funnel. The filtrate was freeze-dried using a freeze dryer and weighed to determine extraction yield according to the following equation: Extraction yield (% *w*/*w*) = (weight of lyophilized extract (g)/weight of dried plant material (g)) × 100. The dried extract was then stored in airtight, light-protected containers at −20 °C to preserve its stability and bioactivity.

### 2.3. Determination of AC Bioactive Compounds

All phytochemical analyses, including total phenolic content (TPC), total flavonoid content (TFC), total tannin content (TTC), and verbascoside quantification, were performed using three independent extract samples, with each assay conducted in triplicate. TPC was determined using the microplate Folin–Ciocalteu method [28]. A sample solution (1 mg/mL) was prepared in water. A 20 µL aliquot was mixed with 100 µL of Folin–Ciocalteu reagent and 80 µL of 1 M Na_2_CO_3_. The mixture was incubated at room temperature for 10 min, and absorbance was measured at 630 nm using a 96-well microplate reader. Results were expressed as mg gallic acid equivalents per gram of extract (mg GAE/g extract). The TFC value was determined using the aluminum chloride colorimetric method, as modified from Nicolescu et al. (2025) [29]. A 50 µL aliquot of sample (1 mg/mL in water) was mixed with 50 µL of 5% NaNO_2_ and incubated for 5 min, followed by the addition of 20 µL of 10% AlCl_3_. After 6 min, 100 µL of 1 M NaOH was added, and the mixture was allowed to stand for 30 min. Absorbance was measured at 510 nm. Rutin (Sigma, London, UK) was used as the standard, and the results were expressed as milligrams of rutin equivalents per gram of extract (mg RE/g extract). The TTC value was determined using the Folin–Ciocalteu method [30]. A 0.2 mL aliquot of the sample (1 mg/mL) was mixed with 2.5 mL of distilled water and 0.2 mL of Folin–Ciocalteu reagent, followed by the addition of 2 mL of 7% Na_2_CO_3_. After incubation at room temperature for 90 min, absorbance was measured at 748 nm. Results were expressed as milligrams of tannic acid equivalents per gram of dry extract (mg TAE/g). Additionally, verbascoside content was quantified by HPLC-UV [31] using a C18 column (150 × 4.6 mm, 5 µm). The mobile phase consisted of solvent A (acetonitrile) and solvent B (0.1% formic acid in water), with the following gradient program: 0–5 min, 10–16% A; 5–10 min, 16–25% A; 10–25 min, 25–38% A; and 25–30 min, 38–10% A. The flow rate was 0.8 mL/min, the column temperature was maintained at 30 °C, and the injection volume was 10 µL. Detection was performed at 240 nm. Verbascoside standard (purity ≥ 95%) was purchased from Sigma-Aldrich (St. Louis, MO, USA). The verbascoside content was calculated from a standard calibration curve and expressed as % (*w*/*w*).

### 2.4. Antioxidant Activity, Cell Viability and Anti-Inflammatory Activity

All antioxidant assays (DPPH, ABTS, and FRAP) were conducted using three independent extract samples, and each measurement was performed in triplicate to ensure analytical reliability. Measurements of DPPH radical-scavenging activity were performed according to Nara et al. (2006) [32]. Sample solutions (25–500 µg/mL) were prepared in water. A volume of 20 µL of each sample was mixed with 180 µL of 0.1 mM DPPH solution in a 96-well plate, incubated in the dark at room temperature for 15 min, and the absorbance was measured at 517 nm. Radical-scavenging activity (%) was calculated, and IC_50_ values were determined from the concentration–response curve. The ABTS radical scavenging assay was performed using previously reported methods Payet et al. (2005) [33]. ABTS^+^ was generated from 4 mM ABTS and 4.9 mM K_2_S_2_O_8_ (1:1), incubated in the dark for 16 h and diluted to absorbance 0.7 ± 0.2 at 734 nm. A 20 µL sample (100–1500 µg/mL) was mixed with 280 µL of ABTS working solution, incubated at 30 °C for 5 min, and the absorbance was measured at 734 nm. IC_50_ values were calculated.

The FRAP activity was assessed based on the previously reported protocol of Benzie and Strain (1999) [34]. FRAP reagent was prepared from acetate buffer (pH 3.6), 10 mM TPTZ in HCl, and 20 mM FeCl_3_·6H_2_O (10:1:1). A 20 µL sample (10 µg/mL) was mixed with 150 µL FRAP reagent and incubated. Absorbance at 593 nm was read at 0 and 4 min. A FeSO_4_ calibration curve (0.1–1.0 mM) was used to calculate antioxidant activity as mmol Fe^2+^/g extract.

Anti-inflammatory activity was evaluated in LPS-induced RAW264.7 macrophages [35]. Cells (1000 cells/µL) were seeded in 96-well plates and incubated at 37 °C, 5% CO_2_ for 24 h. LPS (1 µg/mL final) and extracts (25–200 µg/mL) were added. The solvent control received DMEM only (aqueous extract). After 24 h, nitrite production in supernatants was measured using the Griess assay [36] at 540 nm. Cell viability was assessed by MTT assay at 570 nm. The percentage inhibition of nitric oxide (NO) production and percentage cytotoxicity were calculated using the following formulas:

% inhibition = [(Absorbance control) − (Absorbance treatment)]/(Absorbance control)] × 100

### 2.5. Experimental Shrimp and Trial Procedures, Along with Sample Collection

**Figure 1:**
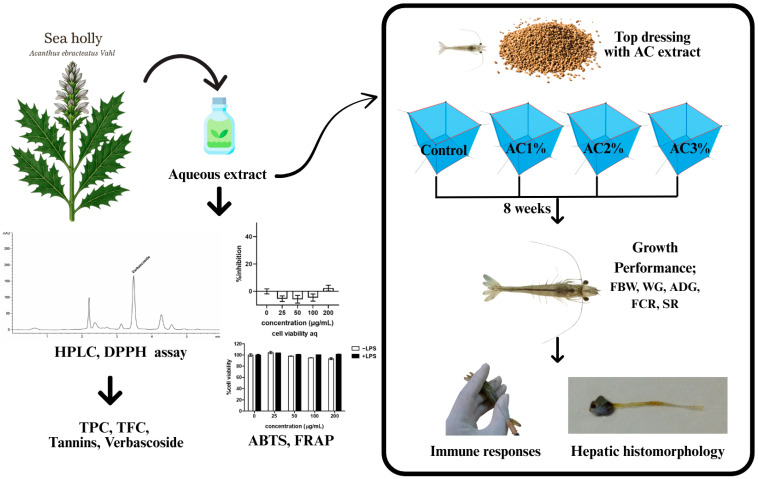
The study map of the effects of AC aqueous extract on Pacific white shrimp (*L. vannamei*).

Healthy Pacific white shrimp post-larvae (2.00 ± 0.01 g initial body weight) were obtained from a commercial shrimp farm in Chanthaburi Province, Thailand. Shrimp were first acclimated for one week in a 1-ton aerated tank and fed the basal diet. After acclimation, 300 shrimp were randomly distributed into 12 cages (1 m × 1 m × 1 m), corresponding to a stocking density of 25 shrimp/m^2^. The experimental groups were assigned to four dietary treatments: Control, AC 1%, AC 2%, and AC 3% with three replicates per treatment (Figure 1).

The feeding trial was conducted for 8 weeks in a static-water system with continuous aeration. An 8-week feeding period was selected because it is widely used in shrimp nutrition studies and provides sufficient time to evaluate the effects of dietary supplementation on growth performance, feed utilization, immune responses, antioxidant status, and hepatopancreatic health in juvenile Pacific white shrimp. Shrimp were manually fed three times daily (08:00, 13:00, and 18:00 h). The initial feeding rate was 5% of total biomass per day and was adjusted weekly based on the measured biomass of each cage. Feed amounts were reduced proportionally as shrimp grew, following standard commercial practice for Pacific white shrimp. Uneaten feed was collected 30 min after each meal by siphoning, dried, and weighed to estimate actual feed intake. Water quality was maintained within optimal ranges throughout the study. Each cage received 10–20% daily water exchange, depending on turbidity and ammonia levels. Dissolved oxygen was kept above 5.0 ± 0.2 mg/L through continuous aeration, and water parameters were monitored daily, including temperature (28.3 ± 0.3 °C), pH (7.9 ± 0.1), salinity (11 ± 0.3 ppt), and ammonia (<0.5 mg/L). Throughout the experimental period, no disease outbreaks, abnormal mortality events, or feed-related problems were observed in any treatment group. In addition, all monitored water quality parameters remained within acceptable ranges for Pacific white shrimp culture, indicating that environmental conditions were stable throughout the trial. At the end of the 8-week trial, shrimp were fasted for 12 h and then collectively weighed to evaluate growth performance and feed utilization. Hemolymph and hepatopancreas samples were subsequently collected from randomly selected individuals as previously described [37]. For immunological analysis, hemolymph collected from 10 shrimp in each cage was pooled to form a single composite sample per cage (n = 3 per treatment). For histological evaluation, hepatopancreas samples were analyzed individually from three randomly selected shrimp per cage (n = 9 per treatment). Each cage was considered the experimental unit (n = 3 per treatment), and all statistical analyses were performed using cage means.

### 2.6. Diet Preparation and Proximate Analysis

The experiment was designed as a completely randomized design (CRD) consisting of four treatments with three replicates each. Feed supplementation was applied based on the results of our previous studies, which demonstrated significant improvements in growth performance and enhanced immunostimulatory responses [38]. In this study, a commercial diet was used as the basal diet. According to the manufacturer’s formulation, the basal diet did not contain added herbal extracts, phytogenic feed additives, probiotics, or other functional bioactive supplements. Therefore, the control diet served as an appropriate reference for evaluating the effects of AC supplementation. All experimental diets were formulated to be isonitrogenous and isolipidic, closely following the nutrient requirements of Pacific white shrimp [39]. The experimental design comprised four dietary treatments for Pacific white shrimp: a control group receiving the basal diet and three treatment groups supplemented with AC extract at inclusion levels of 1% (AC 1%), 2% (AC 2%), and 3% (AC 3%), respectively. All treatment diets, the AC extract was dissolved in water and then coated with binder, the drying under room temperature for two hours and keep the diets in refrigerator. The proximate composition of the experimental feeds, including crude protein, crude lipid, moisture, and ash, was analyzed following standard procedures outlined by AOAC (2012) [40]. Moisture content was determined by oven-drying at 105 °C, ash content by incineration in a muffle furnace at 550 °C, crude protein (N × 6.25) using the Kjeldahl method with a Kjeltec 2300 Analyzer (Foss Tecator, Höganäs, Sweden), and crude lipid by ether extraction using a Soxtec System HT6 (Tecator). The results are presented in Table 1. Based on the quantified phytochemical composition of the AC extract, the estimated dietary concentrations of total phenolics, total flavonoids, and verbascoside supplied by each supplementation level were calculated and are presented in Supplementary Table S1.

### 2.7. Evaluation of Growth Performance, Feed Utilization, and Morphological Parameters

All shrimp were fasted for 12 h prior to final sampling and weighing. The final wet weight of each shrimp was individually recorded, and the number of surviving shrimps in each cage was counted. Growth performance parameters, including initial body weight (IBW), final body weight (FBW), weight gain (WG), average daily gain (ADG), feed conversion ratio (FCR), and survival rate (SR), were evaluated to assess the effects of sea holly extract. These parameters were calculated after the 8-week feeding trial based on the methods described by Abbas et al. (2023) [41], as detailed in Equations (1)–(4):

WG (g) = FBW − IBW(1)

ADG (g/shrimp/day) = (FBW − IBW)/days(2)

SR (%) = [(Number of shrimp harvested/Number of shrimps stocked)] × 100(3)

FCR = Dry feed intake/(FBW − IBW)(4)

### 2.8. Evaluation of Shrimp Immunological Parameters

All antioxidant and immune-related parameters were determined using laboratory-prepared reagents according to previously published spectrophotometric methods. At the end of the experimental period, hemolymph (10 shrimp per cage) was collected using a sterile syringe preloaded with anticoagulant solution. The anticoagulant consisted of sodium citrate and NaCl prepared at a ratio of 1:1 (*v*/*v*), and hemolymph were mixed with anticoagulant at a volume ratio of 1:1 (*v*/*v*) immediately during collection. The samples were stored at −20 °C until biochemical analysis. The innate immune response parameters were evaluated in shrimp hemolymph. Catalase (CAT) activity was measured following the method of Weydert and Cullen (2010) [42] by mixing 10 µL of shrimp hemolymph with H_2_O_2_ and measuring the absorbance at 240 nm using a microplate reader (VersaMax™, Molecular Devices, San Jose, CA, USA). Superoxide dismutase (SOD) was measured following Misra and Fridovich (1977) [43] by mixing 20 µL of shrimp hemolymph with sodium carbonate buffer (pH 10.2) and epinephrine, then measuring at 490 nm. Glutathione peroxidase (GPx) was assessed following the protocol of Fontagné-Dicharry et al. (2020) [44] by mixing 20 µL of shrimp hemolymph with solutions containing Triton X-100 (Sigma, UK) and glutathione, incubating, adding H_2_O_2_, and measuring at 450 nm. Total antioxidant content (T-AOC) was determined using the DPPH radical scavenging assay following Wangkahart et al. (2023) [45] by mixing with 180 μL of 0.2 mM DPPH in ethanol and incubated in the dark at 30 °C for 30 min and measuring at 520 nm. The concentration of hemocyanin was investigated as previously described with a little modification Pan et al. (2019) [46] by mixing with 30 μL of shrimp hemolymph and distilled water, and absorbance was measured at 335 nm.

### 2.9. Evaluation of Histomorphology

At the conclusion of the 8-week trial, the middle part of the hepatopancreas samples from shrimps were collected to evaluate the effects of AC on hepatic histomorphology. Hepatopancreas tissues were collected and analyzed individually from three shrimp in each cage, yielding a total of nine independent samples per treatment. These nine biological replicates were used to assess treatment-related differences in hepatopancreatic morphology. Histological examination aimed to confirm signs of tissue atrophy and structural irregularities in the hepatopancreas, which are characteristic pathological features associated with acute hepatopancreatic necrosis disease (AHPND). Three samples shrimp per group were subjected to a 24 h fasting period prior to the collection of hepatic samples. In brief, the collected hepatopancreas samples were transferred from 10% neutral buffered formalin to a 4% paraformaldehyde solution for 48 h. The hepatopancreas samples were then dehydrated through a graded series of ethanol concentrations, ranging from 70% to absolute ethanol, cleared in xylene, and impregnated and embedded in paraffin wax, in accordance with the protocol described by Wangkahart et al. (2023) [45]. The hepatopancreas tissues were sectioned at a thickness of 5 μm using a semiautomatic microtome. Histological sections were stained with Harris’ hematoxylin and eosin (H&E), with each staining step conducted for 5 min and followed by rinsing in distilled water [47] and visualized under a light microscope (Nikon DS-Ri2, Boston, MA, USA) for morphological analysis and images were captured at 40× magnification.

### 2.10. Synchrotron Radiation-Based Fourier Transform Infrared (SR-FTIR) Spectroscopy

Hepatopancreas samples were prepared using a paraffin-embedding technique. The embedded tissues were sectioned at approximately 4 µm using a microtome and mounted on IR-reflective slides (Mirr-IR Corner Frosted, Kevley Technologies, Chesterland, OH, USA). The sections were then deparaffinized and dried prior to analysis. Spectra were acquired using a Tensor 27 FTIR spectrometer (Bruker Optics, Ettlingen, Germany) coupled with a Hyperion 2000 IR microscope equipped with a 36× objective and a mercury cadmium telluride (MCT) detector. FTIR spectra were recorded in reflection mode with 64 scans at a resolution of 6 cm^−1^ over the spectral range of 4000–800 cm^−1^. Spectral acquisition and instrument control were performed using OPUS 7.5 software (Bruker Optics Ltd., Ettlingen, Germany). Spectra from each group were analyzed using principal component analysis (PCA) to differentiate biochemical components among tissue samples. PCA was used as an exploratory multivariate approach to visualize spectral variation and clustering among treatments. Statistical comparisons among treatment groups were performed using the relative integral areas of selected spectral regions. These variables were analyzed by one-way ANOVA followed by Duncan’s multiple range test, with significance accepted at *p* < 0.05. Data preprocessing included baseline correction and vector normalization using the Savitzky–Golay method (third-order polynomial, 13 smoothing points) in UnscramblerX 10.1 software (CAMO, Bergen, Norway). Three-dimensional (3D) score and loading plots were used to visualize dataset variation.

### 2.11. Statistical Analysis

Data were analyzed using one-way ANOVA followed by Duncan’s multiple range test, after verifying normality (Shapiro–Wilk test) and homogeneity of variances (Levene’s test). Orthogonal polynomial contrasts were applied to assess linear and quadratic effects of AC inclusion levels. When these assumptions were not met, appropriate data transformations were performed. Because survival rate is proportional data and typically deviates from normality, survival values were subjected to an arcsine square-root transformation prior to analysis. ANOVA was then conducted on the transformed survival data, while untransformed means (%) are presented in the tables for clarity. Statistical significance was set at *p* < 0.05. All analyses were performed using IBM SPSS Statistics version 20 (SPSS Inc., Chicago, IL, USA).

## 3. Results

### 3.1. Phytochemical Profiles

The phytochemical composition of AC, including phenolic compounds, flavonoids, tannins, and verbascoside, is presented in Table 2. The aqueous extract of sea holly yielded a brown powder with an extraction yield of 3.32% (*w*/*w*). The extract exhibited a high content of total phenolic compounds (162.1 ± 11.78 mg GAE/g extract), total flavonoids (75.8 ± 2.32 mg RE/g extract), and total tannins (38.4 ± 0.78 mg TAE/g extract). The quantification of verbascoside revealed 0.48 ± 0.01% *w*/*w*.

### 3.2. Assessment of Cell Viability and Antioxidant Activity

The anti-inflammatory effect of the aqueous extract was evaluated in LPS-stimulated RAW264.7 macrophages by measuring NO production. At concentrations ranging from 25 to 200 µg/mL, the aqueous extract did not exhibit significant inhibition of NO production when compared to the LPS-treated control group. However, cell viability assays demonstrated that the aqueous extract was non-cytotoxic at all tested concentrations. The viability of RAW264.7 cells remained above 90% compared to untreated controls, indicating that the extract is safe for use at concentrations applied in this study.

The antioxidant activities of AC aqueous extract were assessed using three standard in vitro assays, including DPPH, ABTS, and FRAP, as illustrated in Table 2. The DPPH assay results showed that the extract showed an IC_50_ value of 42.6 ± 11.78 µg/mL, indicating potent free radical scavenging activity. The ABTS assay further supported this finding with an IC_50_ of 2.93 ± 0.02 mg/mL. In the FRAP assay, AC aqueous extract demonstrated strong ferric reducing antioxidant power, with a FRAP value of 1.41 ± 0.08 mmol FeSO_4_/g extract.

### 3.3. Growth Performance, Feed Utilization, and Body Condition Indices

The effects of AC supplementation in aquafeed formulations for Pacific white shrimp on growth performance and feed utilization specifically FBW, WG, ADG, SR, and FCR are summarized in Table 3. At the end of the feeding trial, significant differences were observed among treatments in FBW, WG, ADG, and FCR (*p* < 0.001). Shrimp fed the AC 2% diet achieved the highest final body weight (10.06 ± 0.06 g), weight gain (8.06 ± 0.06 g), and ADG (0.13 ± 0.00 g/shrimp/day), all of which were significantly greater than the other treatments. In contrast, both the control and AC 3% groups exhibited significantly reduced values for the growth parameters. A similar trend was observed for FCR, with the AC 2% group demonstrating the most efficient feed utilization (1.20 ± 0.02), significantly outperforming all other treatments, while the control group had the highest FCR (1.79 ± 0.06), indicating the least efficient feed conversion. Orthogonal polynomial contrast analysis revealed significant quadratic effects of AC supplementation on FBW, WG, ADG, and FCR (*p* < 0.001), indicating a non-linear dose–response relationship. Growth performance improved progressively with increasing AC inclusion up to 2%, whereas further supplementation to 3% did not result in proportional improvements. These findings suggest that dietary AC exerted its greatest growth-promoting effect at intermediate inclusion levels. Additionally, survival rates tended to be higher in the AC-supplemented groups (94.67–97.33%) than in the control group (89.33%). However, the overall treatment effect was not statistically significant according to one-way ANOVA (*p* = 0.084). Orthogonal polynomial contrast analysis revealed a significant linear trend (*p* = 0.022), suggesting a tendency for survival to increase with increasing dietary AC inclusion. Therefore, while AC supplementation may have contributed to improved survival, these findings should be interpreted cautiously and require further validation under larger-scale experimental conditions.

### 3.4. Innate Immune Responses

**Figure 2:**
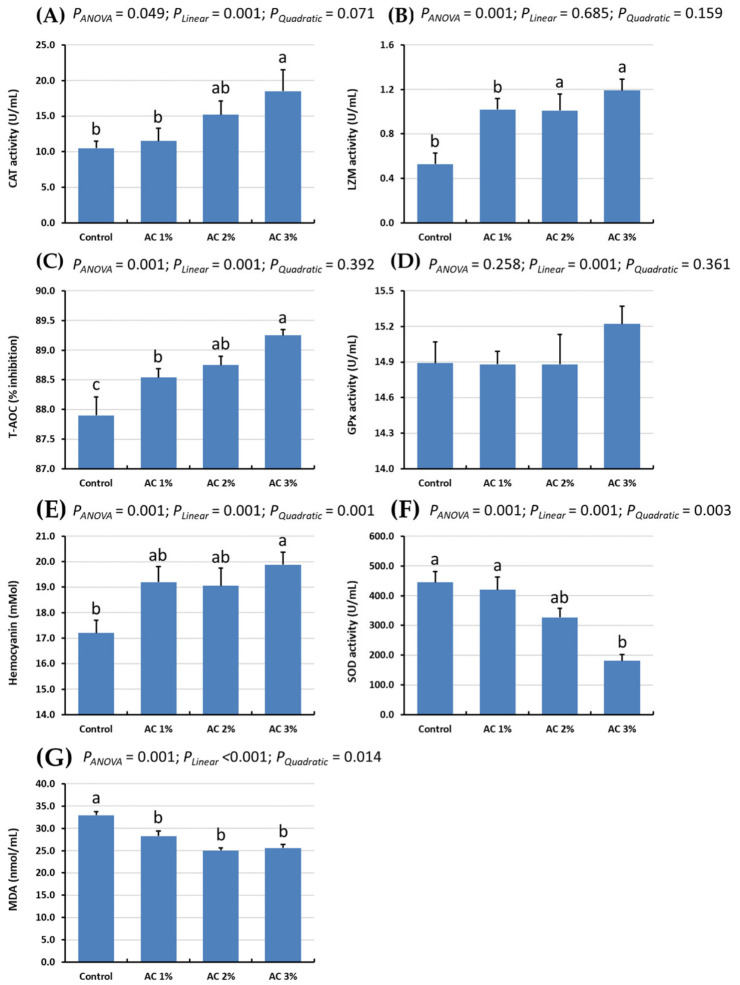
Non-specific immune responses and antioxidant enzyme activities of Pacific white shrimp (*L. vannamei*) after 8 weeks of dietary supplementation with AC extract are presented as follows: (**A**) catalase (CAT) activity; (**B**) lysozyme (LZM) activity; (**C**) total antioxidant capacity (T-AOC); (**D**) glutathione peroxidase (GPx); (**E**) hemocyanin; (**F**) superoxide dismutase (SOD); (**G**) malondialdehyde (MDA). Data are expressed as mean  ±  SEM. Different letters above the bars indicate statistically significant differences (*p*  <  0.05).

The effects of AC supplementation in aquafeed formulations on non-specific immune parameters in Pacific white shrimp are presented in Figure 2. Shrimp fed the diet containing AC 3% exhibited significantly higher activities of CAT, LZM, T-AOC, and hemocyanin compared to the control group (*p* < 0.05). It should be noted that hemocyanin primarily functions as an oxygen transport protein, and its potential immunological role requires further confirmation. In contrast, SOD activity was significantly lower in shrimp fed the AC 3% diet than in the control group (*p* < 0.05). Additionally, LZM activity was significantly increased in all treatment groups compared to the control group (*p* < 0.05). A significant decrease in MDA was also observed in shrimp receiving AC-supplemented diets compared to the control group (*p* < 0.05).

### 3.5. Histological Parameters

**Figure 3:**
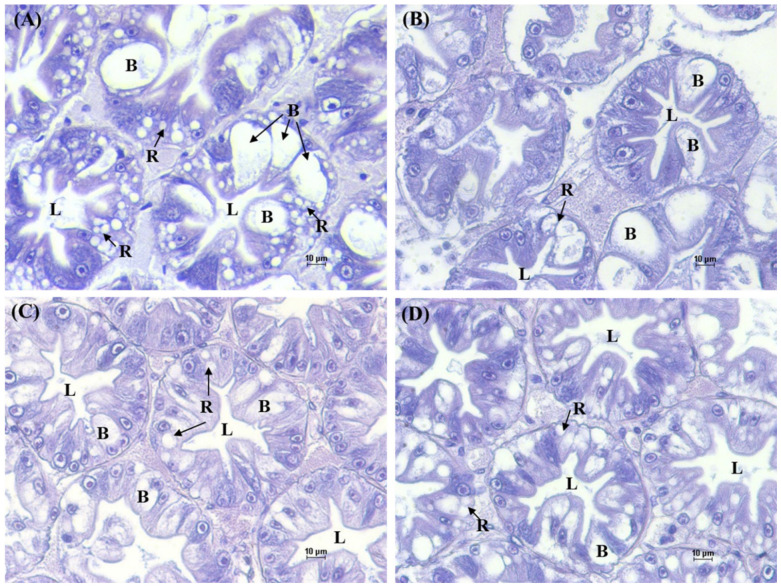
Photomicrographs of hepatopancreatic sections (magnification ×40) from Pacific white shrimp after 8 weeks of dietary supplementation with AC extract are presented: (**A**) control diet, (**B**) AC 1%, (**C**) AC 2%, and (**D**) AC 3% supplementation. Structural features such as the lumen (L) of the hepatopancreatic tubule, restzellen cells (R), and B-cells (B; secretory cells) are indicated. Arrows (←) indicate the locations of restzellen cells (R) and B-cells (B). Images were captured using a light microscope with a 40× objective lens (total magnification 400×). Scale bar = 10 μm.

Morphological structure of the hepatopancreas in Pacific white shrimp fed experimental diets supplemented with sea holly extract is presented in Figure 3. The hepatopancreas of shrimp in the control group exhibited normal tissue organization, including the presence of B-cells with characteristic cytoplasmic vacuoles and R-cells containing visible lipid droplets, which are common features of healthy shrimp hepatopancreatic tissue. In contrast, the AC 1% supplementation group demonstrated moderate improvements, including enhanced tubule compactness and more uniform cellular distribution. The most pronounced histological improvement was observed in the AC 2% group, which displayed well-organized tubules with tightly packed structures, consistent B- and R-cell arrangement, and regular luminal morphology. Histological examination revealed mild vacuolization and structural disorganization in the hepatopancreas of shrimp fed the AC 3% diet, although overall tissue integrity remained intact. These results demonstrate that the AC 2% diet induced the most notable improvements in hepatopancreatic morphology, exhibiting well-organized cellular arrangements indicative of enhanced tissue function and physiological status.

### 3.6. FTIR Signatures of Hepatopancreas Associated with AC Extract

**Figure 4:**
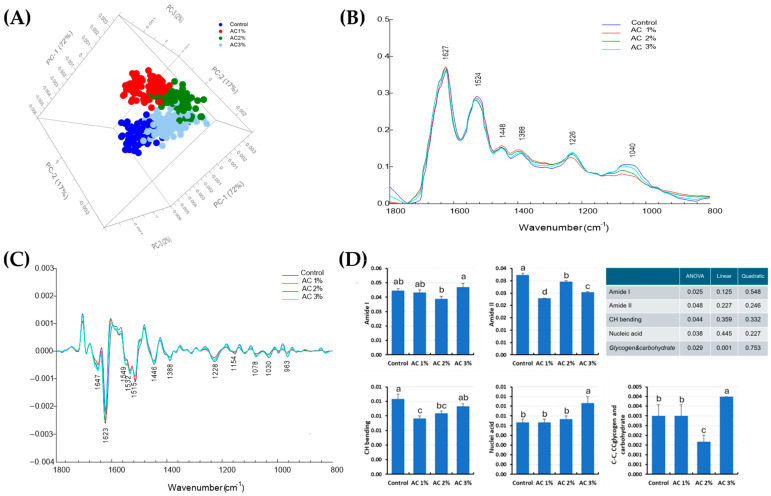
FTIR-based biomolecular characterization of lumen tissue from shrimp hepatopancreas under different dietary treatments. (**A**) PCA score plot showing spectral clustering among treatments. (**B**) Average original spectra (4000–800 cm^−1^). (**C**) Second-derivative spectra highlighting subtle vibrational differences. (**D**) Relative integral area of major biomolecular regions: Amide I, Amide II, CH bending, nucleic acids, and glycogen/carbohydrates, different lowercase letters above the bars indicate statistically significant differences among treatment groups (*p* < 0.05). All measurements collected at 50 × 50 µm, 6 cm^−1^ resolution, 64 scans, ≥150 spectra per group.

FTIR spectroscopy can sensitively detect the biochemical characteristics of cellular macromolecules such as proteins, lipids, nucleic acids, and carbohydrates, providing insights into cellular organization and physiological states [48,49]. In this study, distinct spectral changes were observed in the biomolecular composition of shrimp hepatopancreas in the lumen tissue of the hepatopancreas (Figure 4), the spectral characteristics of biomolecular components showed clear variations in the Amide I protein peak (1700–1600 cm^−1^). However, no pronounced differences were detected among the treatment groups, and no statistically significant differences were observed at the *p* < 0.05 level. In contrast, the AC 3% group exhibited the most notable alterations in nucleic acid-associated peaks (1240 and 1080 cm^−1^) and in the glycogen/carbohydrate region (1200–900 cm^−1^), with these differences being statistically significant at *p* < 0.05. With respect to protein secondary structures, the lumen tissue primarily displayed a β-sheet conformation at 1621 cm^−1^, which was most abundant in the AC 1%, AC 2%, and control groups, and least evident in the AC 3% group. Conversely, the α-helix structure at 1650 cm^−1^ was generally less prominent, but occurred at higher levels in the AC 1% and AC 3% groups, and was least apparent in the AC 2% group, with the control group showing similarly low levels.

## 4. Discussion

The application of herbal plant extracts is widely recognized for providing multiple benefits to aquatic animals, particularly shrimp, including improvements in growth performance, immune function, antioxidant capacity, and resistance to disease [15,50,51]. AC, in particular, contains various bioactive compounds such as phenolic acids, flavonoids, iridoids, and O-glycosylated compounds that have shown potential as immunostimulants in shrimp [24]. These bioactive substances can be administered in the form of whole plants, specific plant parts, or extracted compounds and are commonly applied as aqueous solutions incorporated into feed additives. A thorough understanding of the mechanisms of action of medicinal plants, especially AC, is essential for optimizing their targeted and effective application in shrimp aquaculture. To the best of our knowledge, documentation on the application of AC as a feed additive in aquaculture remains limited, particularly with respect to its potential effects on growth performance, histological alterations, and immune modulation in aquatic animals. Therefore, the present study investigated the phytochemical composition, antioxidant potential, and biofunctional impacts of AC extract to elucidate its mechanisms as a dietary supplement in shrimp aquaculture for nutritional enhancement and health management.

The bioactivity of medicinal plant extracts is largely governed by their phytochemical composition, with the presence and relative abundance of specific bioactive constituents playing a critical role in determining their functional efficacy [24]. Numerous natural substances have demonstrated an abundance of pharmacological properties, including the ability to influence oxidative stress through strong antioxidant pathways. These include isolated molecules and crude plant extracts. In this study, the phytochemical profiling of AC aqueous extract revealed a diverse array of bioactive compounds, including phenolic compounds, flavonoids, tannins, and the phenylethanoid glycoside verbascoside. The extract, obtained as a brown powder, exhibited notably high levels of total phenolics (162.1 ± 11.78 mg GAE/g extract), flavonoids (75.8 ± 2.32 mg RE/g extract), and tannins (38.4 ± 0.78 mg TAE/g extract). These findings are consistent with previous studies reporting the abundance of polyphenolic constituents in AC [20,24], which are known to exert potent antioxidant, anti-inflammatory, and antimicrobial activities. The high levels of phenolics and flavonoids suggest that AC may serve as an effective phytogenic additive in aquaculture, capable of modulating antioxidant defense systems and improving health parameters in shrimp. Particularly noteworthy is the quantification of verbascoside (0.48 ± 0.01% *w*/*w*), a major phenylethanoid glycoside with well-documented pharmacological properties. Verbascoside has been associated with free radical scavenging, inhibition of pro-inflammatory cytokines, and protective effects against oxidative stress in various biological systems [52]. The substantial presence of this compound in the extract underscores its potential functional role in promoting cellular protection and enhancing immunophysiological responses in aquatic species. These Phytochemical profiles likely contribute synergistically to the observed physiological benefits, including enhanced tissue morphology and hepatopancreatic integrity, as demonstrated in the present study. The chemical profile of the extract thus provides a mechanistic basis for its bioactivity and supports its application as a functional ingredient in shrimp feed formulations.

The aqueous decoction of AC stems and leaves yielded 3.32% (*w*/*w*) dry extract, which, while modest, is consistent with previously reported yields for aqueous preparations of AC and related mangrove species [24], where water-soluble extraction methods inherently recover a narrower fraction of total phytochemical constituents compared to organic solvent-based approaches. Despite the relatively low yield, the extract exhibited notably high concentrations of phenolics, flavonoids, tannins, and verbascoside, confirming that the aqueous decoction selectively enriched bioactive water-soluble constituents with demonstrated antioxidant and immunostimulatory properties. From a practical perspective, a 3.32% yield implies that approximately 30 kg of dried plant material is required to produce 1 kg of extract. Although this warrants consideration in commercial contexts, the high biological potency observed at dietary inclusion levels of 1–3% means that the absolute quantity of extract required per tonne of feed remains practically manageable. The use of renewable aerial parts, stems and leaves, further supports scalability, as these can be harvested without destroying the plant. Future work optimizing extraction parameters such as decoction time, temperature, and solid-to-liquid ratio may improve yield while maintaining bioactive integrity. In addition, the application of advanced extraction techniques, such as ultrasound-assisted extraction, microwave-assisted extraction, or pressurized liquid extraction, may significantly improve extraction yield and bioactive recovery, thereby further enhancing the commercial feasibility of AC extract as a large-scale functional aquafeed additive.

The antioxidant capacity of AC aqueous extract was systematically assessed through three standard in vitro assays DPPH, ABTS, and FRAP which collectively evaluated its free radical scavenging ability and ferric reducing power, thereby reflecting its phytochemical richness and potential pharmacological significance. In the DPPH radical scavenging assay, the extract demonstrated an IC_50_ value of 42.6 ± 11.78 µg/mL, indicating a strong ability to neutralize stable free radicals. This level of activity is comparable to those reported for other medicinal plants with high phenolic and flavonoid contents, suggesting that the observed effect may be attributed to the synergistic action of these compounds [53,54]. Furthermore, the ABTS assay, which evaluates the ability of antioxidants to quench the ABTS^+^ radical cation, further supported the extract’s radical scavenging capacity, yielding an IC_50_ value of 2.93 ± 0.02 mg/mL. Although slightly higher than the DPPH result, this finding is consistent with the extract’s broad-spectrum antioxidant properties and may reflect differences in assay sensitivity and radical species [55,56]. FRAP assay was employed to assess the ferric reducing antioxidant power of the extract. The sea holly extract exhibited a FRAP value of 1.41 ± 0.08 mmol FeSO_4_/g extract, indicating a high electron-donating capacity. This strong reducing potential further confirms the antioxidant efficacy of the extract and underscores its ability to act as a reductant in oxidative environments. Earlier studies by Aktaruzzaman et al. (2025) [24] indicated that the leaf extract of AC exhibited significantly higher antioxidant activity in DPPH (19.99 µg/mL TE/g), ABTS (2.71 mg/mL TE/g), and FRAP (1.11 mmol FeSO_4_ eq/g extract) compared to extracts from the stem and root, suggesting a greater efficacy in free radical scavenging and metal ion reduction. In comparison, our study also confirmed notable antioxidant activity in the leaf extract; however, the observed DPPH, ABTS, and FRAP values were substantially higher, indicating an even greater antioxidant potential under the conditions applied. Altogether, these findings demonstrate that AC possesses significant antioxidant activity, likely attributable to its high content of phenolic compounds, flavonoids, and other redox-active constituents. Such antioxidant capacity is of particular relevance in aquaculture, where oxidative stress can impair physiological performance and immune function. The results therefore support the potential application of AC extract as a functional feed additive to enhance antioxidant defense mechanisms in shrimp.

Anti-inflammatory potential of the AC aqueous extract was assessed in LPS-stimulated RAW264.7 macrophages by quantifying NO production, a key pro-inflammatory mediator. Across the investigated concentration range (25–200 µg/mL), the extract did not produce a statistically significant reduction in NO levels compared to the LPS-treated control, suggesting limited anti-inflammatory activity under the conditions of this assay. The absence of significant anti-inflammatory activity may be related to the aqueous extraction method used in the present study. Aqueous extraction primarily recovers hydrophilic compounds and may not efficiently extract lipophilic or semi-polar constituents responsible for anti-inflammatory activity. Previous studies reporting anti-inflammatory effects of AC commonly employed ethanolic or organic solvent extracts, which differ substantially in phytochemical composition from aqueous decoctions. Therefore, the limited NO inhibitory activity observed here likely reflects solvent-dependent extraction efficiency rather than the absence of anti-inflammatory constituents in the plant. Nevertheless, the extract demonstrated strong antioxidant properties and good biocompatibility. Cell viability assays indicated that the aqueous extract was non-cytotoxic at all tested concentrations, with RAW264.7 macrophage viability consistently exceeding 90% relative to untreated controls. These results support the potential application of the extract in biological systems, particularly where antioxidant activity and safety are prioritized. Earlier reports have also described additional pharmacological properties of AC, including wound-healing, neuroprotective, and antimicrobial activities [31,57]. Furthermore, the findings of the present study are consistent with Wisuitiprot et al. (2022) [31], who similarly reported that aqueous extracts of AC showed limited anti-inflammatory activity in macrophage assays compared with ethanolic extracts. Collectively, these results suggest that the beneficial effects of the aqueous AC extract observed in shrimp are more likely associated with antioxidant modulation than with direct anti-inflammatory activity under the tested conditions. A limitation of this study is that no pharmacological positive control was included in the LPS-RAW264.7 NO inhibition assay. Therefore, although AC extract did not significantly inhibit nitric oxide production, the responsiveness of the assay system could not be independently verified. Future studies should include an appropriate positive control to validate assay performance and strengthen interpretation of the anti-inflammatory results.

Our study demonstrates, for the first time, the potential of AC leaf and stem extracts as a functional feed additive in Pacific white shrimp aquaculture, with the results indicating that dietary AC supplementation significantly enhances growth performance and feed utilization in this species. Shrimp fed diets supplemented with AC 2% exhibited the most pronounced improvements in FBW, WG, ADG, and FCR, with all parameters showing statistically significant differences, indicating growth performance, nutrient utilization and metabolic efficiency in shrimp. This dose-dependent response is consistent with findings from other studies, where excessive levels of phytochemicals may disrupt gut microbial balance or inhibit digestive enzymes, ultimately impairing nutrient absorption. Notably, both the control and AC 3% groups recorded significantly lower values across these growth parameters, suggesting that excessive inclusion levels may negatively affect growth outcomes. In terms of feed utilization, the AC 2% diet demonstrated the most efficient feed conversion, with the lowest FCR, significantly outperforming all other treatments. In contrast, the basal diet exhibited the highest FCR, indicating suboptimal feed efficiency. The phytochemical analysis of the AC extracts revealed the presence of key bioactive compounds, especially polyphenol, which are known for their antioxidant and immunomodulatory properties. Although no previous studies have directly evaluated AC leaf extracts in aquaculture species, related research has documented the pharmacological properties of this plant in mammals. For instance, Prasansuklab and Tencomnao (2018) [20] reported that AC root extracts stimulated murine macrophage activity and upregulated the expression of immune-related genes. Overall, these findings highlight the beneficial effects of AC 2% on growth performance, and survival in Pacific white shrimp, supporting its potential use as a functional feed additive in shrimp aquaculture. The improvements in growth performance observed in shrimp fed the 2% AC diet may be mechanistically linked to enhanced digestive and metabolic efficiency. Polyphenols and flavonoids present in AC are known to stimulate digestive enzyme secretion, modulate gut microbial composition, and improve nutrient assimilation [14,25]. We hypothesize that the high phenolic content of the extract enhanced proteolytic and lipolytic activity in the hepatopancreas, thereby supporting more efficient feed conversion and nutrient storage. This is consistent with previous studies demonstrating that phytogenic additives rich in phenolics improve growth performance and FCR in Pacific white shrimp and other aquaculture species through similar mechanisms [11,27].

The innate immune response, which constitutes the first line of defense against microbial infections, is triggered by the recognition of microbe-associated molecular patterns (MAMPs) through host pattern recognition receptors (PRRs), leading to the activation of intracellular signaling pathways and the induction of both cellular and humoral immune mechanisms. In the present study, we found that dietary AC 2% provide an optimal balance for nutrient utilization, digestive efficiency, and hepatopancreatic function, thereby supporting growth performance. In contrast, the 3% AC supplementation level may provide a greater concentration of bioactive compounds, thereby more effectively stimulating antioxidant defenses and innate immune responses in shrimp. A comprehensive understanding of shrimp immunology is therefore critical for the development of effective disease prevention and control strategies in shrimp aquaculture [58], demonstrating that dietary supplementation with AC at 3% markedly enhances the non-specific immune responses in Pacific white shrimp. Notably, shrimp receiving the AC 3% diet exhibited significantly elevated levels of CAT, LZM, T-AOC, and hemocyanin, indicating a strong activation of the innate immune system. These findings are consistent with previous reports suggesting that herbal bioactive can enhance immunological functions in crustaceans through the modulation of antioxidant and antimicrobial defense systems [11,50,59].

It should be noted that prior immunostimulatory investigations of AC have predominantly employed root extracts [19]. We acknowledge that phytochemical composition can differ substantially among roots, stems, and leaves of AC, which may influence their biological activities and limit direct comparison with previous root-based immunostimulatory studies. Therefore, the biological effects observed in the present study should be interpreted in the context of the specific phytochemical characteristics of the stem and leaf aqueous extract used herein, particularly its richness in phenolics, flavonoids, and verbascoside. These differences in phytochemical composition may contribute to distinct antioxidant and physiological effects compared with those previously reported for root extracts. In the present study, stems and leaves were selected on the basis of their documented abundance of phenolics, flavonoids, and verbascoside [20,24], the ecological sustainability of harvesting aerial parts from mangrove habitats, and their greater practicality for large-scale aquafeed production. Notably, Prasansuklab and Tencomnao (2018) [20] reported that AC extracts possess strong antioxidant and antimicrobial activities, likely associated with their high phenolic and flavonoid contents. Although organ-specific differences in phytochemical composition limit direct comparison with root-derived immunostimulatory findings, the significant enhancements in CAT, LZM, T-AOC, and hemocyanin observed here support the functional value of leaf and stem extracts as a renewable source of bioactive feed additives for shrimp aquaculture.

The increase in T-AOC and CAT activities further underscores the potential of AC to mitigate oxidative stress in shrimp. These enzymes play critical roles in neutralizing reactive oxygen species (ROS), thereby maintaining cellular redox balance. The antioxidant potential of AC extract is attributed to its rich content of polyphenolic compounds, which are suggested to confer protective effects against oxidative stress-induced damage [24]. The observed reduction in MDA levels across all AC-supplemented groups provides additional evidence of decreased lipid peroxidation, suggesting that AC may exert protective effects on cellular membranes under culture-related stress conditions. Interestingly, although CAT activity was elevated in all AC-supplemented groups, a significant reduction in SOD activity was observed only in the AC 3% group. Therefore, increased CAT activity alone may not fully explain the observed SOD response. One possible explanation is that the highest dietary inclusion level induced a distinct antioxidant adjustment that was not triggered at lower supplementation levels. Under this scenario, the coordinated activity of downstream antioxidant enzymes may have reduced the requirement for sustained SOD activity once oxidative balance was effectively maintained. However, because antioxidant signaling pathways, reactive oxygen species production, and gene expression profiles were not evaluated in the present study, this interpretation should be regarded as a hypothesis rather than a confirmed mechanism [60]. Similar compensatory trends have been reported in shrimp and fish exposed to functional dietary additives with strong antioxidant properties [61,62]. The consistent enhancement of LZM activity in all AC-supplemented groups highlights the immunostimulatory properties of AC. As a key component of the shrimp’s humoral immune defense, LZM contributes to the degradation of bacterial cell walls, thus playing a vital role in protecting against pathogenic invasion. The observed upregulation of hemocyanin, a multifunctional protein involved in oxygen transport and immune defense, further supports the immunopotentiation effect of AC supplementation. In crustaceans, hemocyanin primarily functions as an oxygen transport protein, although previous studies have also reported its involvement in innate immune defense, including antimicrobial activity, phenoloxidase-like activity, and participation in pathogen recognition pathways. Collectively, these findings indicate that dietary inclusion of AC, particularly at 3%, can significantly improve immune function and oxidative status in Pacific white shrimp. The enhanced immune-related responses observed in AC-fed shrimp suggest a potential improvement in physiological preparedness against stressors and pathogens. However, because no pathogen challenge trial was conducted, the extent to which these responses translate into enhanced disease resistance remains unknown. Further studies evaluating long-term performance, immune gene expression, disease challenge trials, THC, prophenoloxidase activity, and other cellular immune parameters are needed to better confirm the immunomodulatory role of AC supplementation in Pacific white shrimp under commercial culture conditions. The enhanced immune responses, particularly in the AC 3% group, suggest a dose-dependent immunostimulatory effect. The elevated activities of CAT, T-AOC, and LZM indicate that the extract enhances both enzymatic antioxidant defenses and humoral immunity. Polyphenolic compounds can activate Nrf2-mediated antioxidant pathways, leading to the upregulation of cytoprotective enzymes [52]. We therefore hypothesize that AC supplementation modulated redox homeostasis by activating intracellular signaling pathways that improve the shrimp’s capacity to neutralize ROS. Similar immune-enhancing effects have been documented in shrimp fed other herbal extracts such as *A. ilicifolius*, *Eleutherine bulbosa*, and *Alpinia oxyphylla* [26,51,55]. The contrasting decrease in SOD activity observed in the AC 3% group may reflect a compensatory antioxidant mechanism. When CAT activity is elevated, the physiological demand for SOD may decrease, as hydrogen peroxide (H_2_O_2_) is rapidly processed downstream [60,62]. This hypothesis aligns with antioxidant enzyme interdependency reported in crustaceans exposed to dietary phytogenic stimulants. SOD converts superoxide radicals into H_2_O_2_, which is subsequently detoxified by CAT and GPx. In the AC 3% group, elevated CAT activity together with increased GPx activity may have enhanced H_2_O_2_ clearance, thereby reducing the physiological demand for SOD. This coordinated antioxidant response may explain the reduced SOD activity despite enhanced antioxidant defenses. This interpretation is further supported by the significantly lower MDA levels in AC-supplemented groups, indicating reduced lipid peroxidation and effective control of oxidative stress through CAT- and GPx-mediated detoxification pathways. However, the higher inclusion level may also slightly increase metabolic cost or physiological burden, which could explain why immune-related parameters peaked at 3% while growth did not further improve beyond 2%. Thus, the results suggest a dose-dependent trade-off, where moderate AC supplementation optimizes growth, whereas a higher dose preferentially stimulates immune and antioxidant responses.

Hepatopancreas is a vital multifunctional organ in shrimp, serving as the principal component of the digestive system, a central site for metabolic processes, and a primary source of immune-related molecules [63]. Structurally, it comprises paired tubular glands lined with epithelial cells specialized for food digestion, nutrient absorption, glycogen and lipid storage, as well as the synthesis of innate immune effectors and the regulation of hormonal metabolism. Theses study highlights the modulatory effects of AC extract on the hepatopancreatic morphology of Pacific white shrimp. In this study, we also detected in control group, which received no supplementation, exhibited notable histopathological alterations, including disorganized tubule architecture, vacuolated B-cells, and lipid-laden R-cells. These features are commonly associated with hepatocellular stress, impaired lipid metabolism, and compromised tissue integrity, consistent with previous observations in shrimp subjected to suboptimal dietary or environmental conditions. Dietary inclusion of AC 1% led to moderate histological improvement, as evidenced by increased tubule compactness and a more uniform distribution of epithelial cells. This suggests a partial restoration of tissue structure, potentially linked to the antioxidant and anti-inflammatory properties of phytochemicals present in AC. The most significant enhancement was observed in the AC 2%, which exhibited a well-organized hepatopancreatic histoarchitecture, characterized by tightly packed tubules, consistent alignment of B- and R-cells, and regular luminal morphology. These features may be attributed to the functional role of B-cells, which serve as the primary site for digestive enzyme synthesis, reflecting a physiologically active hepatopancreas and suggesting that the AC 2% supplementation level effectively supports tissue integrity and cellular homeostasis [64]. Whereas, consistent alignment of R-cells, reflects as efficient nutrient absorption and metabolism, stable intracellular homeostasis, and minimal oxidative or inflammatory stress [65]. Interestingly, the AC 3% did not exhibit further histological improvement; instead, mild vacuolization and structural irregularities reappeared, although overall tissue integrity remained preserved. This inverse response at higher supplementation levels may indicate a threshold beyond which the extract no longer provides additional benefits and may even induce mild cellular stress. Similar dose-dependent effects have been reported in studies evaluating phytogenic additives in aquatic species, where moderate inclusion levels promote physiological health, while excessive doses may disrupt cellular balance [11,66,67]. In summary, these results suggest that dietary supplementation with 2% AC extract confers the most favorable effects on hepatopancreatic histology in Pacific white shrimp, likely through mechanisms involving the modulation of oxidative stress, enhancement of lipid metabolism, and preservation of epithelial cell architecture. These findings provide valuable insights into the dose-dependent effects of herbal additives on shrimp health and support the application of AC as a functional ingredient in aquafeed formulations. Hepatopancreatic improvements in the AC 2% group suggest that AC phytochemicals promote structural integrity of digestive tubules and reduce cellular stress. Well-organized B- and R-cells imply efficient digestion and lipid metabolism, respectively. We hypothesize that AC supplementation mitigates oxidative stress–induced tissue damage, thereby preserving hepatopancreatic architecture. These findings agree with recent studies demonstrating that plant-derived antioxidants prevent vacuolization and epithelial deterioration in shrimp hepatopancreas during thermal, salinity, or pathogen-induced stress [47,63]. The dose-dependent decline in histological quality at 3% AC suggests a threshold beyond which beneficial effects plateau or reverse. Excess phytochemicals may cause mild cytotoxicity or disrupt metabolic equilibrium an effect also reported for other herbal additives in shrimp and fish [66,67]. Thus, the 2% inclusion level appears to provide an optimal balance between physiological benefit and biological tolerance. To the best of our knowledge, these mechanistic insights and comparisons with previous research support the conclusion that AC possesses strong potential as a phytogenic additive capable of improving health, immunity, and hepatopancreatic function in Pacific white shrimp. Further research should focus on validating these hypotheses through targeted assays such as digestive enzyme profiling, gut microbiota characterization, oxidative stress biomarkers, and pathogen challenge trials. From a practical perspective, the use of 2% supplementation appears to provide an optimal balance between growth performance and immune enhancement, making it a promising candidate for application in commercial shrimp farming systems.

A limitation of the present study is that hepatopancreatic morphology was assessed qualitatively without quantitative morphometric measurements or histopathological scoring. Although representative micrographs suggested treatment-related differences, future studies should incorporate objective histological indices to provide statistically robust evaluation. In addition, the economic feasibility, scalability, and long-term effects of AC supplementation under commercial farming conditions remain to be determined. While AC improved several immune-related parameters, no pathogen challenge trial was conducted, and key immune indicators such as THC, phagocytic activity, and prophenoloxidase activity were not evaluated. Therefore, the observed immunomodulatory effects should be interpreted cautiously, and future studies should include both cellular and humoral immune assessments, together with pathogen challenge tests, to better characterize the protective potential of AC supplementation.

## 5. Conclusions

The present study provides the first comprehensive evaluation of AC aqueous extract as a functional feed additive in Pacific white shrimp. The extract, characterized by high levels of phenolics, flavonoids, tannins, and verbascoside, demonstrated strong antioxidant capacity and exerted measurable benefits on shrimp physiology. Dietary supplementation at 2% produced the most favorable effects on growth performance, feed conversion, and hepatopancreatic morphology, while the 3% inclusion level most strongly enhanced key nonspecific immune responses. Although the present study used 0%, 1%, 2%, and 3% AC extract inclusion levels as an initial screening design, future studies should include additional intermediate doses, such as 0.5%, 1.5%, and 2.5%, to better characterize the dose–response relationship and more precisely determine the optimal inclusion level for balancing growth performance and immune enhancement. Together, these findings highlight the dose-dependent biofunctional properties of AC and support its potential as a natural, plant-derived additive that can contribute to improved health and productivity in shrimp aquaculture. Beyond its biological effects, the use of locally abundant mangrove plants such as AC also aligns with sustainable aquaculture goals by reducing reliance on synthetic compounds and antibiotics. This study therefore contributes new evidence supporting the integration of phytogenic additives into modern shrimp farming practices. Future research should extend these findings by investigating long-term feeding responses under commercial farming conditions, evaluating pathogen-challenge performance to confirm disease-protective effects, and exploring additional mechanisms such as modulation of gut microbiota, metabolic pathways, and antioxidant gene expression. Further work is also warranted to evaluate the scalability, cost-effectiveness, and stability of AC extract in pelleted feed for large-scale aquaculture operations. AC extract, particularly at a 2% inclusion level, effectively enhanced growth performance, immune responses, and hepatopancreatic health in Pacific white shrimp, supporting its application as a natural additive in shrimp aquaculture. Nevertheless, these findings were obtained under controlled conditions and should be further validated in commercial aquaculture settings. The practical application of AC supplementation requires further validation through pathogen challenge studies and commercial-scale production trials to confirm its effectiveness under farming conditions.

## Figures and Tables

**Table 1:** Proximate composition of the experimental diets (dry weight basis).

Proximate Composition	Control	AC 1%	AC 2%	AC 3%
Moisture (%)	10.47	11.15	11.59	13.26
Ash (%)	10.48	10.33	10.46	10.37
Crude lipid (%)	7.51	7.19	6.94	6.69
Crude protein (%)	37.02	37.68	37.65	36.61
Fiber (%)	1.87	1.75	2.03	1.74

**Table 2:** Phytochemical and antioxidant activities of AC aqueous extracts.

Parameters	AC Aqueous Extracts
Quantitative analysis of phytochemical constituents	
Total phenolic content (mg gallic acid per g extract)	162.1 ± 11.78
Total flavonoid content (mg rutin per g extract)	75.8 ± 2.32
Total tannin content (mg tannic acid per g extract)	38.4 ± 0.78
Verbascoside (% *w*/*w*)	0.48 ± 0.01
Antioxidant capacity	
IC_50_ of DPPH assay (µg/mL)	42.6 ± 11.78
IC_50_ of ABTS assay (mg/mL)	2.93 ± 0.02
FRAP assay (mmol Fe_2_SO_4_/g extract)	1.41 ± 0.08

**Table 3:** Growth performance and body condition parameters of Pacific white shrimp under four dietary treatments for 8 weeks.

–	Control	AC 1%	AC 2%	AC 3%	*p*-Value		
					ANOVA	Linear	Quadratic
Initial body weight (g)	2.00 ± 0.02	2.00 ± 0.02	2.00 ± 0.01	2.01 ± 0.01	0.986	0.587	0.538
Final body weight (g)	8.29 ± 0.07 ^a^	8.97 ± 0.24 ^b^	10.06 ± 0.06 ^c^	8.19 ± 0.02 ^a^	<0.001	0.208	<0.001
Weight gain (g)	6.29 ± 0.09 ^a^	6.97 ± 0.25 ^b^	8.06 ± 0.06 ^c^	6.18 ± 0.02 ^a^	<0.001	0.197	<0.001
ADG (g/shrimp/day)	0.11 ± 0.00 ^a^	0.12 ± 0.00 ^b^	0.13 ± 0.00 ^c^	0.10 ± 0.00 ^a^	<0.001	0.054	<0.001
Survival rate (%)	89.33 ± 1.33	94.67 ± 3.53	97.33 ± 1.33	97.33 ± 1.33	0.084	0.022	0.241
FCR	1.79 ± 0.06 ^d^	1.46 ± 0.01 ^b^	1.20 ± 0.02 ^a^	1.65 ± 0.04 ^c^	<0.001	0.004	<0.001

Abbreviations: Average daily gain (ADG) and Feed conversion ratio (FCR). Values are presented as means ± SEM. Values within the same row with different superscripts are significantly different (*p* < 0.05).

## Data Availability

The data are available upon request from the corresponding author.

## Supplementary Materials

The following supporting information can be downloaded at: https://www.mdpi.com/article/10.3390/ani16121842/s1, Table S1: Estimated concentrations of major bioactive compounds supplied by dietary AC supplementation.
