# Supplementary material for: Effects of Acanthus ebracteatus (Sea Holly) Aqueous Extract as a Functional Feed Additive on Growth Performance, Immune Responses, and Hepatopancreatic Histology in Pacific White Shrimp (Litopenaeus vannamei)
Source: Animals (Basel). 2026 Jun 15;16(12):1842. doi: 10.3390/ani16121842 (PMC13295723; doi:10.3390/ani16121842)
Supplement: Supplementary file 1 [file animals-16-01842-s001.zip › animals-4302602-supplementary.pdf]

Table S1. Estimated concentrations of major bioactive compounds supplied by dietary AC supplementation.

Parameter	AC 1%	AC 2%	AC 3%
Total phenolics (mg GAE/kg feed)	1,621	3,242	4,863
Total flavonoids (mg RE/kg feed)	758	1,516	2,274
Verbascoside (mg/kg feed)	48	96	144

Values were calculated from the measured phytochemical composition of the AC extract and the dietary inclusion level. Concentrations represent estimated bioactive compound delivery and were not directly measured in the finished feeds.
